# Deficits in Visuo-Motor Temporal Integration Impacts Manual Dexterity in Probable Developmental Coordination Disorder

**DOI:** 10.3389/fneur.2018.00114

**Published:** 2018-03-05

**Authors:** Satoshi Nobusako, Ayami Sakai, Taeko Tsujimoto, Takashi Shuto, Yuki Nishi, Daiki Asano, Emi Furukawa, Takuro Zama, Michihiro Osumi, Sotaro Shimada, Shu Morioka, Akio Nakai

**Affiliations:** ^1^Neurorehabilitation Research Center, Kio University, Nara, Japan; ^2^Graduate School of Health Science, Kio University, Nara, Japan; ^3^Department of Rehabilitation, Higashi Osaka Yamaji Hospital, Osaka, Japan; ^4^Department of Rehabilitation, Nanso-no-Sato, Nursing Care Insurance Facilities, Osaka, Japan; ^5^Department of Rehabilitation, Nogami Hospital, Osaka, Japan; ^6^Department of Home-Visit Rehabilitation, Ishida Clinic, Osaka, Japan; ^7^Department of Rehabilitation, Japan Baptist Hospital, Kyoto, Japan; ^8^Faculty of Education, Kio University, Nara, Japan; ^9^Department of Electrical Engineering, Graduate School of Science and Technology, Meiji University, Kanagawa, Japan; ^10^Department of Electronics and Bioinformatics, School of Science and Technology, Meiji University, Kanagawa, Japan; ^11^Hyogo Children’s Sleep and Development Medical Research Center, Kobe, Japan

**Keywords:** autism-spectrum disorder, attention-deficit hyperactivity disorder, automatic imitation, developmental coordination disorder, internal model, manual dexterity, mirror-neuron system, visuo-motor temporal integration

## Abstract

The neurological basis of developmental coordination disorder (DCD) is thought to be deficits in the internal model and mirror-neuron system (MNS) in the parietal lobe and cerebellum. However, it is not clear if the visuo-motor temporal integration in the internal model and automatic-imitation function in the MNS differs between children with DCD and those with typical development (TD). The current study aimed to investigate these differences. Using the manual dexterity test of the Movement Assessment Battery for Children (second edition), the participants were either assigned to the probable DCD (pDCD) group or TD group. The former was comprised of 29 children with clumsy manual dexterity, while the latter consisted of 42 children with normal manual dexterity. Visuo-motor temporal integration ability and automatic-imitation function were measured using the delayed visual feedback detection task and motor interference task, respectively. Further, the current study investigated whether autism-spectrum disorder (ASD) traits, attention-deficit hyperactivity disorder (ADHD) traits, and depressive symptoms differed among the two groups, since these symptoms are frequent comorbidities of DCD. In addition, correlation and multiple regression analyses were performed to extract factors affecting clumsy manual dexterity. In the results, the delay-detection threshold (DDT) and steepness of the delay-detection probability curve, which indicated visuo-motor temporal integration ability, were significantly prolonged and decreased, respectively, in children with pDCD. The interference effect, which indicated automatic-imitation function, was also significantly reduced in this group. These results highlighted that children with clumsy manual dexterity have deficits in visuo-motor temporal integration and automatic-imitation function. There was a significant correlation between manual dexterity, and measures of visuo-motor temporal integration, and ASD traits and ADHD traits and ASD. Multiple regression analysis revealed that the DDT, which indicated visuo-motor temporal integration, was the greatest predictor of poor manual dexterity. The current results supported and provided further evidence for the internal model deficit hypothesis. Further, they suggested a neurorehabilitation technique that improved visuo-motor temporal integration could be therapeutically effective for children with DCD.

## Introduction

Developmental coordination disorder (DCD), which is characterized by an inability to perform age-appropriate fine (hand writing and shoelace tying) and gross motor skills (playing sport and getting dressed) (1), affects approximately 6% of school-aged children, making it the most common childhood movement disorder (1). Such a broad range of deficits not only impacts performance of daily tasks, but also contributes to secondary long-term health consequences, including reduced engagement in physical activity and social activities (2, 3), and increased risk of low self-esteem, anxiety, and depression (3, 4). Coordination difficulties seen in 50–70% of children with DCD persist into adolescence and adulthood (1). Although the behavioral motor impairments experienced by children who have DCD are well known, the etiology, and neurological origin that has been long suspected to contribute to such deficits, underlying these impairments remain unclear (5). Among the motor impairments seen in DCD, we specifically focused on clumsy manual dexterity in the current study. Further, we evaluated the internal model deficit (6–9) and mirror-neuron system (MNS) deficit (10, 11), i.e., two putative pathophysiological mechanisms thought to impede fine motor skills in DCD, consequently affecting manual dexterity.

Internal modeling deficits (IMDs) have been proposed as a neurological cause of DCD (12, 13). According to the IMD hypothesis, the sensory–motor integration in the internal model is dysfunctional in children with DCD, which reduces their ability to use predictive motor control (12, 13). Before slow, sensory–motor feedback becomes available, internal models provide stability to the motor system by predicting the outcome of movements. This allows rapid online correction (7, 8). More specifically, during the generation of a motor plan, the motor command is generated by the motor cortex and relayed to the body. An efference copy of this motor command is concurrently generated as a corollary discharge and relayed to the parietal lobe and cerebellum. Then, the predicted and actual sensory feedback is compared with somatic events and visuospatial integration, which are processed at the level of the cerebellum and parietal cortex, respectively. Mismatch between motor predictions (e.g., efference copy, predicted sensory feedback) and actual sensory feedback generates error signals are generated, which correct/modulate the unfolding motor output commands in real time. The parietal cortex and cerebellum is the neural basis for the internal model for online correction (14).

The IMD hypothesis of DCD is supported by several studies that demonstrated deficits in the predictive control of manual action, posture, gait, and eye movements. Paradigms, including the covert orienting of visuospatial attention (15–17), mental rotation of limb-versus object-based stimuli (18–20), grip force and anticipatory postural adjustments (21–23), predictive control of eye movements (24, 25), imagined or simulated pointing (26, 27), and rapid online control of reaching movements (6–9), have been applied to test the IMD hypothesis. The results of these studies converge on the argument that children with DCD have difficulty representing a predictive model of a prospective action, based on the integration of visuospatial/somatic information and motor programming, i.e., sensory–motor integration. Deficits in internal modeling in DCD have also been attributed to dysfunctions in the parietal lobe and cerebellum by previous brain imaging studies (28–34).

However, it has not been clarified whether the time window of visuo–motor integration in the internal model in children with DCD differs from those with typical development (TD). The time window of visuo–motor integration, i.e., the visuo–motor temporal integration ability, can be quantitatively examined using the delayed visual feedback detection task (35). The delay-detection threshold (DDT) and steepness of the probability curve for delay detection, which will simply be referred to as steepness herein, can be determined from this task. The DDT, which is the time delay at which the rate of delay detection is 50%, indicates the extent to which the brain allows temporal discrepancy in different modalities of sensation. The steepness indicates the mechanism by which the brain integrates multisensory signals; a greater steepness indicates a more strict or precise judgment (35). Therefore, a lower DDT and higher steepness represents a highly sensitive visuo–motor temporal integration. Brain imaging and neuromodulation studies revealed that detection of delayed visual feedback for self-generated movement is based on comparison of motor signals (e.g., motor predictions, actual proprioceptive feedback) and actual visual feedback, possibly in the parietal cortex and cerebellum (36–39).

Since previous studies suggested that DCD has an internal model deficit and parietal cortex and cerebellum dysfunction, children with DCD can be expected to have the difficulty with visuo–motor temporal integration. More specifically, the current study hypothesized that DDT was elevated, while steepness was decreased for children with DCD, in comparison to children with TD. Experimental task 1 (the delayed visual feedback detection task) in the present study was designed to examine this hypothesis.

There is recent evidence that MNS deficits are also associated with DCD (10, 11, 40). It is postulated that the human MNS consists of the pars opercularis of the inferior frontal gyrus (IFG), adjacent ventral premotor cortex, and inferior parietal lobule. The MNS is the neural basis of action observation and imitation (41) and plays an integrative role in observational learning. It forms a core circuit for action imitation, aiding learning, and acquisition of new skills *via* modeling others’ behavior and actions (42). Thus, deficits in MNS function interfere with a child’s ability to learn or imitate movements they observe. In fact, several previous studies reported that, compared to children with TD, the performance of imitating meaningful or meaningless intransitive or transitive gestures of children with DCD was significantly reduced (43–47). In addition, an fMRI study conducted by Reynolds et al. (10) reported that the activity of the IFG during action imitation significantly decreased in children with DCD, compared to those with TD. However, the imitation test used in previous studies were adult apraxia assessment batteries, which require advanced cognitive function, and thus, may not be suitable to evaluate a child’s imitation ability (48).

The MNS is responsible for automatic imitation, i.e., the unconscious tendency to mimic the behavior of others (41, 49–52). However, it has not been clarified whether the automatic-imitation function in MNS differs between children with DCD and TD. The automatic-imitation function can be quantitatively investigated using a motor interference task (motor contagion task), which can even be effectively used to assess 4-year-old children (53). In the motor interference task, subjects make sinusoidal right arm movements in the vertical plane while observing another human right arm making either congruent or incongruent movements (i.e., in the vertical or horizontal plane, respectively). The subject experiences interference when observing the incongruent movement executed by the other human. The interference effects (automatic-imitation effects, motor contagion effects) refer to the increased movement fluctuations occurring in the orthogonal (i.e., in the horizontal plane) direction (54). The magnitude of the interference effect has been used as an index of MNS functioning in many previous studies (50, 52, 54–63). In fact, Catmur et al. (64) revealed that disruptive, theta burst transcranial magnetic stimulation of the IFG selectively impaired automatic imitation of abduction movements of the index and little fingers. This previous study provided evidence for the causal relationship between the MNS and automatic imitation.

Since previous studies have suggested that there is dysfunction of the MNS in DCD, children with DCD are expected to have a decline in automatic-imitation function. More specifically, the hypothesis of the current study was that there would be a decline in the interference effect in children with DCD, compared to those with TD. This hypothesis was examined using Experimental task 2 (the motor interference task) in the present study.

Children with DCD often develop depressive symptoms due to motor coordination disorders (65–71). Further, they are also often diagnosed with other developmental disorders, the most common of which is attention-deficit hyperactivity disorder (ADHD), which affects 50% of the population that show both disorders (72–75). Autism-spectrum disorder (ASD) is also indicated as a comorbidity (76–78).

Therefore, the current study cohort was divided into two groups using the manual dexterity test of the Movement Assessment Battery for Children-2nd edition (M-ABC2), which is the international standard evaluation battery for DCD. Since the focus of the current study was on manual dexterity in DCD, the two experimental tasks used evaluated the participants’ upper limbs and hands. The manual dexterity test of the M-ABC2 was used to separate children with probable DCD (pDCD) from those with TD. Children with clumsy manual dexterity were assigned to the pDCD group, while those who were not clumsy were assigned to the TD group. We investigated whether visuo–motor temporal integration ability (Experimental task 1) and automatic-imitation function (self-other visuo–motor integration ability, Experimental task 2) differed between the two groups. In addition, we investigated whether depressive symptoms, ASD traits, and ADHD traits differed among the two groups. Furthermore, correlation and multiple regression analyses were performed to extract factors affecting clumsy manual dexterity.

The purpose of the current study was to investigate factors affecting clumsy manual dexterity, provide useful behavioral markers for understanding the neural mechanism of DCD, and promote the development of new neurorehabilitation techniques to improve clumsy manual dexterity.

## Materials and Methods

### Participants

Participants were recruited from public preschools (i.e., nursery schools and kindergartens), primary schools, and secondary schools, in Osaka, Japan. Children with motor coordination problems were introduced and observed by school teachers and parents. The exclusion criteria included being diagnosed with the following: a general medical condition (e.g., cerebral palsy, muscular dystrophy, hemiplegia, or a degenerative disorder), visual impairment, or intellectual disability {according to criterion D of the DCD diagnostic criteria in the Diagnostic and statistical manual of mental disorders 5th edition [DSM-5 (1)]}. Eligibility was confirmed by interviewing parents and the results of the regular checkup, which was provided by the school doctor at each school. A total of 71 children, with an average age ± SD of 9.8 ± 2.6 years (range: 4.2–15.0 years, boys = 57, right handers = 60) participated in the current study.

Participants were assigned to the pDCD group (*n* = 29, mean percentile ± SD = 4.5 ± 3.5, range: 0.5–9.0 percentile) based on the following: scoring in or below the 15th percentile of the M-ABC2 manual dexterity test; motor coordination problems, which affected activities of daily living and school life, identified by the teachers and parents (criterion B of the DSM-5); and motor coordination problems since early development, identified by the parents (criterion C of the DSM-5). Conversely, even with criterion B and C, participants scoring above the 16th percentile on the M-ABC2 manual dexterity test were assigned to the TD group (*n* = 42, mean percentile ± SD = 53.0 ± 22.7, range: 16.0–84.0 percentile).

There were no significant differences between the groups in terms of sex (pDCD: 25 boys; TD: 32 boys; χ^2^ = 1.087, χ^2^(0.95) = 3.841, *p* = 0.297), age [pDCD: 9.6 ± 2.1 years; TD: 9.9 ± 2.9 years; *t*(68.691) = −0.570, *p* = 0.570], and dominant hand (pDCD: 24 right handers; TD: 36 right handers; χ^2^ = 0.114, χ^2^(0.95) = 3.841, *p* = 0.735). None of the participants had been previously diagnosed with developmental disorder (e.g., ASD, ADHD, learning disorder, pervasive developmental disorder) or depression.

The local ethic committee of the Graduate School and Faculty of Health Sciences at Kio University approved the present study (approval number: H27-33). Parents of the participants provided signed, informed consent.

### Procedures

The manual dexterity test, Experimental task 1 and 2, and the depression self-rating scale for children (DSRS-C) were conducted in each school’s prescribed rooms. The order of implementation of these tasks was randomized for each child. All the tasks were completed in approximately 90 min per child. Concurrently, in another room, the child’s parents responded to the social communication questionnaire (SCQ), ADHD Rating Scale (ADHD-RS), and Developmental Coordination Disorder Questionnaire (DCDQ).

### The Manual Dexterity Test of the Movement Assessment Battery for Children-2nd Edition (M-ABC2)

The manual dexterity test of the M-ABC2 (79) is a standardized, age-adjusted test to identify motor problems in children using different tasks for different age bands. The M-ABC2 had good test retest reliability (minimum value at any age is 0.75), inter-rater value (0.70), and concurrent validity (79). This test has three age bands, which encompasses the following age ranges: 3–6, 7–10, and 11–16 years. In the current study, each child received three sub-tests that were appropriate for their age bands. The sub-tests were as follows: age band 1 (3–6 years), posting coins test, threading beads test, and drawing trail I test; age band 2 (7–10 years), placing pegs test, threading lace test, and drawing trail II test; and age band 3 (11–16 years): turning pegs test, triangle with nuts and bolts test, and drawing trail III test. Based on the examiner’s manual of M-ABC2, the percentile was calculated from the obtained raw scores. The percentile reflected the degree of manual dexterity for each age year, where an increased percentile represented improvement of manual dexterity within each age-group. All the assessments were administrated by a specifically trained, certified physical therapist.

### Experimental Design-1: Delayed Visual Feedback Detection Task

#### Experiment-1: Task

**Figure 1:**
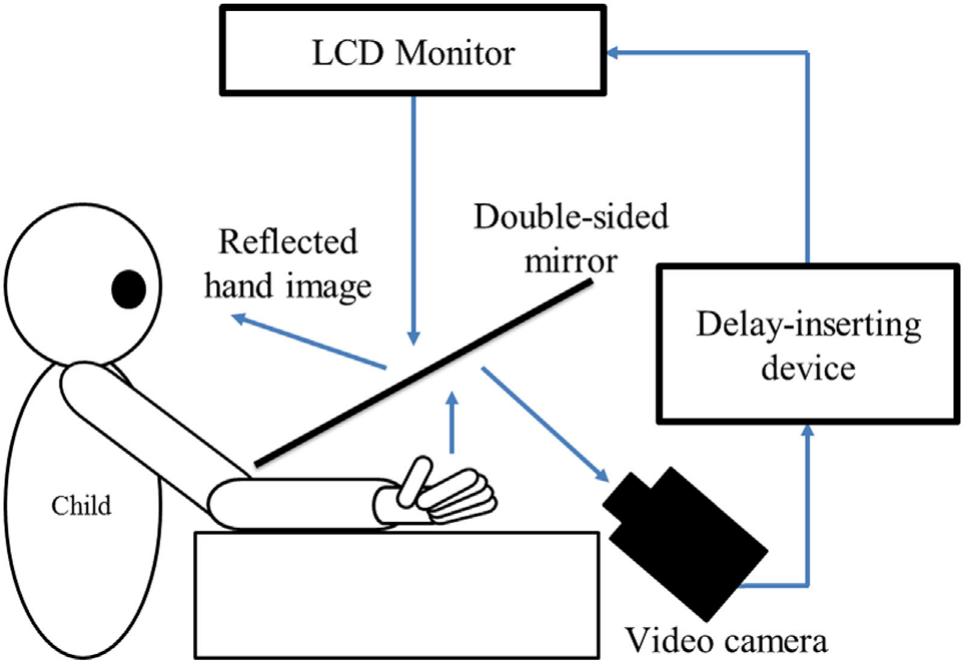
Experiment-1 (Delayed visual feedback detection task). In this study, a similar experimental setup as Shimada et al. (35) was used (Figure 1). The child’s preferred hand was placed under a two-way mirror, so the child was unable to directly see his/her hands. The image of the hand, which was reflected in the two-sided mirror, was filmed with a video camera (FDR-AXP35, Sony, Tokyo, Japan). The movie of the photographed hand was further reflected from an installed monitor (LMD-A240, Sony, Tokyo, Japan) onto the two-sided mirror *via* a video delay-inserting device (EDS-3306, FOR-A YEM ELETEX, Tokyo, Japan). Thus, the child observed the delayed image of their own hand reflected in the mirror at the position where their own hand would be. In addition, the setup included a blackout curtain so that the child would not be able to see outside the experimental chamber. The intrinsic delay of the visual feedback in this experimental setting was 33.7 ms as measured by a time lag check device (EDD-5200, FOR-A YEM ELETEX, Tokyo, Japan).

The delayed visual feedback detection task was performed using the preferred hand of each child (Figure 1). The participants were instructed to observe the reflection in the mirror with the following instruction: “please observe your own hand reflected in the mirror.” Subsequently, the participants opened and closed their hand once, in a continuous and smooth manner, based on the child’s own volition, after the experimenter had orally informed them of the start of a trial. The self-generated movement was regarded when setting the following 18 delay conditions using a video delay-inserting device: 33, 67, 100, 133, 167, 200, 233, 267, 300, 333, 367, 400, 433, 467, 500, 533, 567, and 600 ms. All 18 delay conditions were treated as one set and performed four times; their presentation order was randomized. Consistent with previous studies (35, 39), trials without delay were not included. Thus, each participant completed a total of 72 randomized trials with 18 delay conditions per four sets, which corroborated with previous studies (35, 39).

During the delayed visual feedback detection task, the participants only looked at the reflection of their hand in the mirror, and not their real hand. Thus, the participants could feel their own hand moving while watching the display of the delayed mirror reflection of the same movement. Each participant had to determine if the visual feedback was synchronous or asynchronous, relative to the movement of the preferred hand that was based on their own intention. Immediately following the trial, the participant had to orally state if the movement was “delayed” or “not delayed” by the forced-choice method. A 10-s rest time was set between each trial. A previous study (80), which examined the developmental change of visuo–motor temporal integration ability in children using the same task as in this study, demonstrated that there was no relationship between reaction time/movement speed and visuo–motor temporal integration ability. Thus, the reaction time and movement speed was not recorded in the present study.

The delayed visual feedback detection task was conducted after sufficient explanation and practice to ensure that the children adequately understood the task. Further, before the task, all participants confirmed that they could distinguish between a minimum delay of 33 ms and a maximum delay of 600 ms.

#### Experiment-1: Data Analysis

The logistic curve was fitted to each participant’s response on the visual feedback delay-detection task (35, 81) using the following formula: P(*t*) = 1/1 + exp(−a(*t* − DDT)), where *t* was the visual feedback delay length, P(*t*) was the probability of delay detection, a was the steepness of the fitted curve, and DDT was the observer’s DDT representing the delay length at which the probability of delay detection was 50%. In the current experiment, *t* P(*t*) served as the independent variable and observed value, respectively. The curve was fitted using a nonlinear least squares method (a trust-region algorithm) with the Curve Fitting toolbox in Matlab R2014b (MathWorks Inc., Natick, Massachusetts, USA) to estimate a and DDT.

### Experimental Design-2: Motor Interference Task

#### Experiment-2: Task

**Figure 2:**
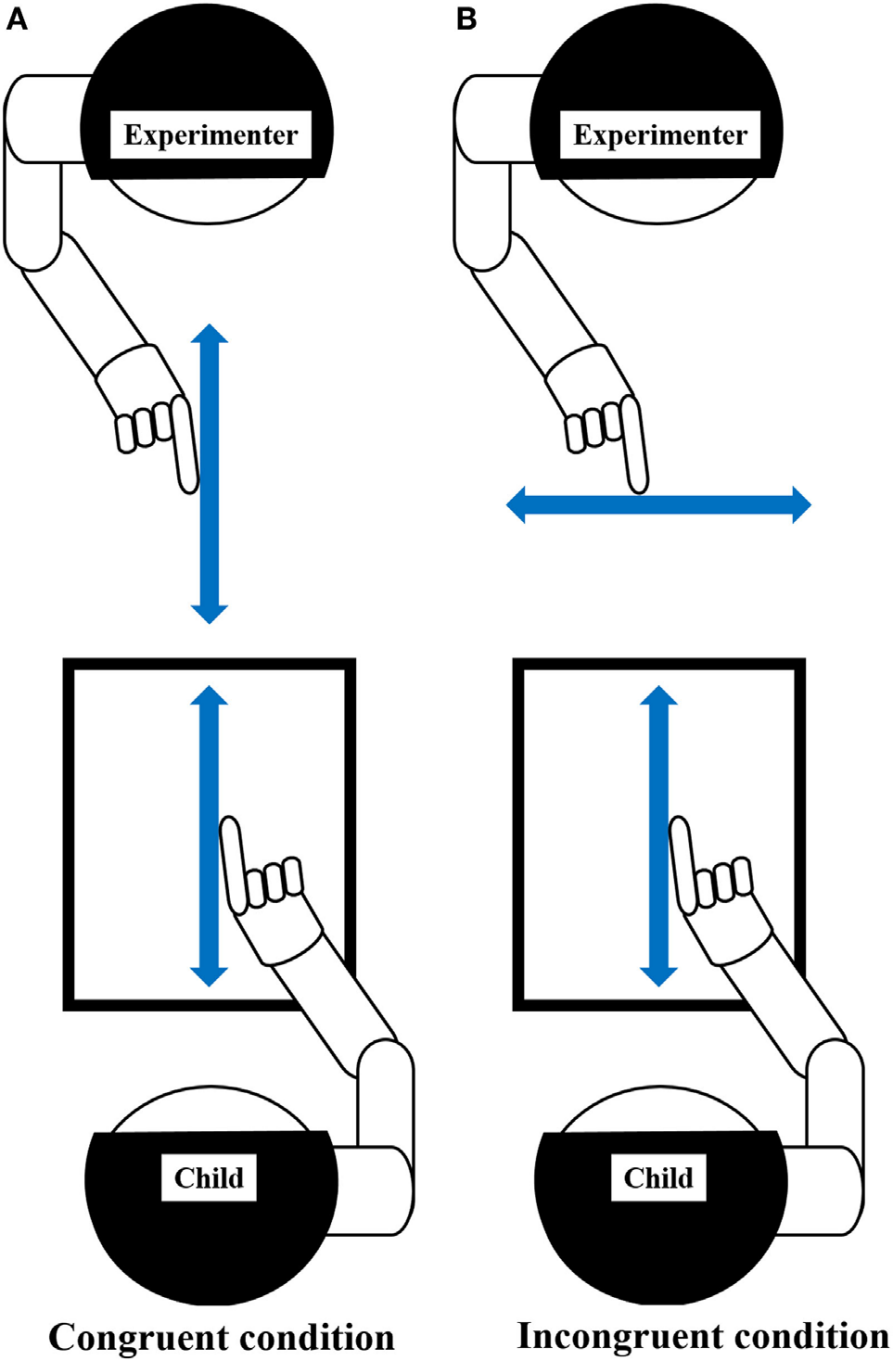
Experiment-2 (Motor interference task). Using the index finger on the preferred hand, the child drew repetitive vertical lines on the Tablet PC, matching the metronome sound of 100 BPM (beats per minute). Arrow, represents the direction of movement. **(A)** Congruent condition: child and experimenter performed repetitive vertical movements. Child repeatedly drew vertical line on the Tablet PC while observing the facing experimenter performing congruent movement (repeating vertical line drawing). **(B)** Incongruent condition: child performed repetitive vertical movement, while experimenter performs repetitive horizontal movement. Child repeatedly drew vertical line on the Tablet PC while observing the facing experimenter performing incongruent movement (repeating horizontal line drawing). Prior to this experiment, the child was given sufficient explanation and carried out enough practice to understand this task. The child demonstrated that they could repeatedly draw a vertical line on the Tablet PC, matching the metronome sound, before starting the trials, even in the absence of the experimenter. This process was repeated as necessary, and the child’s understanding of this task was confirmed. The child had two practice trials for each condition to gain proficiency in the task.

The motor interference task, which was created based on previous studies (53, 54, 59, 82), was conducted as outlined in Figure 2. The task was to draw a vertical line iteratively with a preferred hand’s index finger on the Tablet PC (Surface Pro 4, Microsoft), matching the metronome sound of 100 BPM (beats/min). The drawing distance was 260 mm. For the task, children sat on a chair and installed the Tablet PC on a horizontal desk at an appropriate height. Two conditions, i.e., the congruent and incongruent conditions, were set for the task. In the congruent condition, the child repeatedly drew a vertical line on the Tablet PC while observing the facing experimenter performing the congruent movement (repeatedly drawing a vertical line). In the incongruent condition, the child repeatedly drew vertical line on the Tablet PC while observing the facing experimenter performing an incongruent movement (repeatedly drawing a horizontal).

The experimenter was the same person for the entire cohort and for all trials. The motion trajectory drawn by the child was recorded on the Tablet PC, but was programmed so that the movement locus could not be seen by the child. The child was instructed to repeat their vertical movement, regardless of the experimenter’s movements. The experimenter carried out repetitive vertical line drawing (congruent condition) or repetitive horizontal line drawing (incongruent condition), facing the child. The movement distance of the experimenter was also 260 mm. The experimenter performed the iterative movement using the hand on the same side as the hand used by the child, according to the same 100 BPM metronome sound (e.g., if the child moved the right hand, the experimenter also used the right hand and vice versa). The experimenter closed their eyes during the repetitive movement to avoid being influenced by observing the participant’s movement. The participant conducted two trials for each condition and each trial was 40-s long. The order of trials was randomized. This experimental task was conducted after sufficient explanation and practice to ensure that the participant understood the task.

#### Experiment-2: Data Analysis

The motion trajectory drawn by the participant was analyzed using MATLAB R2014b (MathWorks Inc., Natick, Massachusetts, USA). The first and final five movements of each trial were excluded from analysis. The error value of each reciprocating trajectory was calculated using the following formula: Error value = [SD of vertical-plane data (pixel)/SD of horizontal-plane data (pixel)] × 100 (83, 84). The mean error value for each condition was calculated. An increase in the error value indicated that the participant’s movement trajectory became distorted toward the horizontal direction, while a decrease indicated a more vertical movement trajectory. The interference effect was calculated by subtracting the mean error value of the congruent condition from the mean error value of the incongruent condition (82). An increase in the interference effect indicated an increase in automatic imitation.

### Questionnaires

#### The Depression Self-Rating Scale for Children

Depression in the participants was assessed using the DSRS-C (85, 86), which is a screening test for depression that is composed of 18 items related to the mental condition of the children during the week prior to the test. The scale of the Japanese version has reliable internal consistency (87). Responses were recorded at the following three levels: (1) always; (2) sometimes, and (3) never. The total score ranged from 0 to 36, and a higher score indicated a greater level of depression (87).

#### The Social Communication Questionnaire

The SCQ (88), which is a parent/caregiver report, measures characteristic ASD behaviors, including deficits in social functioning and communication, in children over the age of 4 years. The SCQ includes 40 “yes or no” questions that are derived from the Autism Diagnostic Interview Revised (89). The questions assess the child’s functioning over the past 3 months, as well as at 4–5 years of age. Subscales of the SCQ include the following: communication, reciprocal social interaction, and restricted, repetitive, and stereotyped patterns of behavior. The measure has demonstrated good internal consistency and concurrent validity (88). Higher scores on the SCQ indicate higher levels of autistic traits. The sum of the scores for all 40 questions was calculated to create the total score in the current study.

#### The Attention-Deficit Hyperactivity Disorder Rating Scale

The ADHD-RS-IV (90) is composed of 18 items and scored by a parent/caregiver. Each item corresponds to one of the 18 symptoms in the DSM-IV criteria. Previous research confirmed sufficient reliability and validity for the home form of the ADHD-RS-IV (91). Using a 4-point Likert scale, the children’s parents/guardians rated each item as follows: (0), “not at all or rarely”; (1), “sometimes”; (2), “often”; or (3), “very often.” Therefore, the higher the score and percentile on the ADHD-RS, the more ADHD symptoms the child displayed. This study employed the Japanese version of the ADHD-RS home form (92). The Japanese version of the ADHD-RS demonstrated good reliability and validity (93). In the current study, the sum of the scores for the 18 items was calculated to create a total score (94), and according to the criteria of the Japanese version of the ADHD-RS, the percentile score was calculated from the total score (92).

#### The Developmental Coordination Disorder Questionnaire

The DCDQ is a parent questionnaire designed to screen for pediatric DCD (95, 96). Nakai et al. (97) developed the Japanese version of the DCDQ (DCDQ-J), which is widely applicable to Japanese children (98). The DCDQ-J, which is a 15-item parent rating scale, encompasses the following three factors: general coordination (five items), control during movement (six items), and fine motor skills (four items). Each item is scored using a 5-point scale as follows: (1), “not at all like your child”; (2), “a bit like your child”; (3), “moderately like your child”; (4), “quite a bit like your child”; and (5), “extremely like your child.” Higher scores indicated better motor coordination.

### Statistical Analysis

Inter-group comparisons between the pDCD group and the TD group, and correlation and multiple regression analyses, to detect factors related to clumsy manual dexterity, were performed using SPSS ver. 24 (SPSS, Chicago, IL, USA). The significance level was set at *P* < 0.05.

### Inter- and Intra-Group Comparison

Since the Shapiro–Wilk test demonstrated the normal distribution of DSRS-C scores, an independent *t*-test was used to compare the two groups. However, since the DDT, steepness (Experiment-1), interference effect (Experiment-2), and SCQ, ADHD-RS, and DCDQ scores, were not normally distributed (according to the Shapiro–Wilk test), Mann–Whitney *U* tests were used to compare these measures between the two groups. In addition, a comparison of error values of congruent and incongruent conditions of Experiment-2 was performed using a Wilcoxon signed-rank test.

### Correlation Analysis

A correlation analysis for each measured variable was performed using a Spearman’s correlation coefficient rank test.

### Multiple Regression Analysis

A multiple regression analysis (stepwise method), with manual dexterity (percentile) as the dependent variable and measured items as the independent variables, was performed to extract factors with a significant influence on manual dexterity. Independent variables included DDT/steepness for Experiment-1, error value of the congruent condition for Experiment-2, and the SCQ score, percentile score of ADHD-RS, and DCDQ score.

## Results

### Inter- and Intra-Group Comparison Results

**Figure 3:**
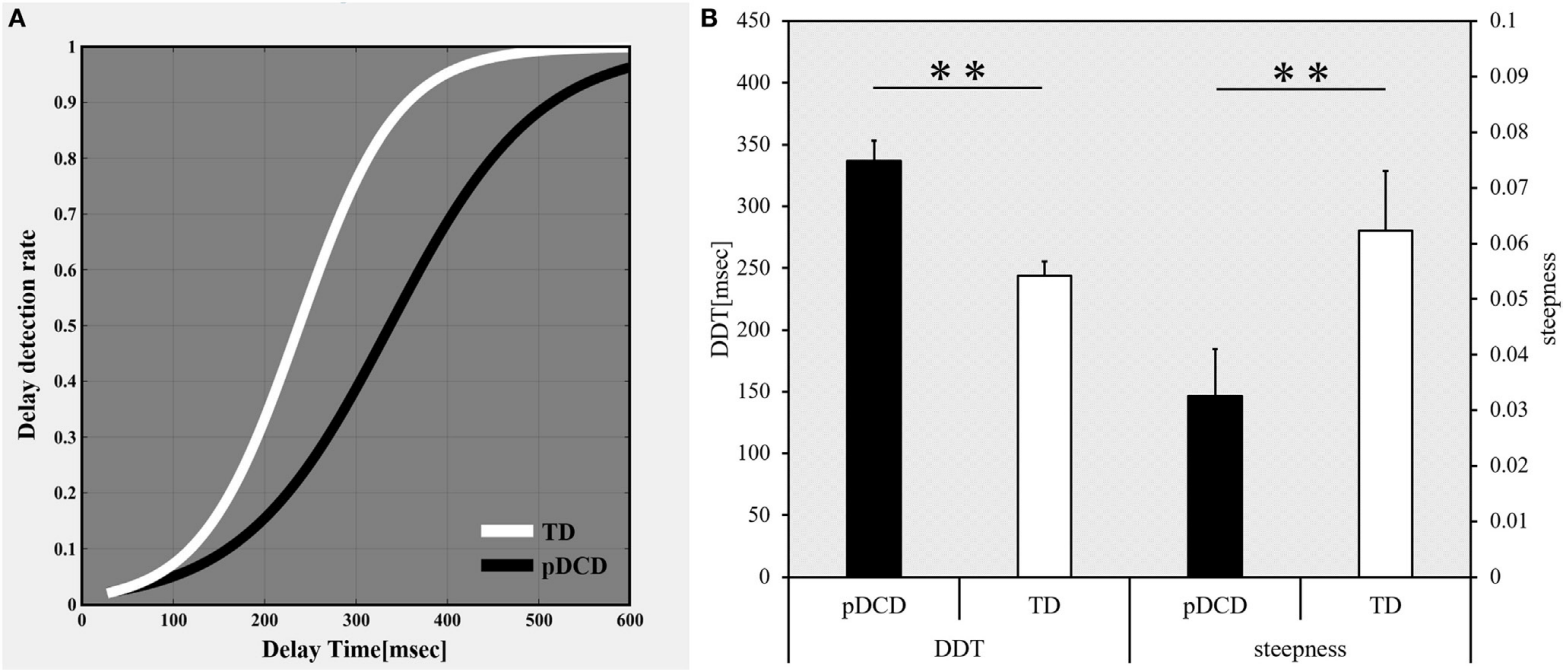
Results of Experiment-1: delayed visual feedback detection task. **(A)** The delay-detection probability curve of each group. White, typical development (TD) group; black, probable developmental coordination disorder (pDCD) group. **(B)** The mean DDT or steepness of each group. Horizontal axis shows each group. Black, pDCD group; white, TD group. Error bars represent the SEM. Left, comparison of the mean DDT of each group; Right, comparison of the mean steepness of each group. DDT, delay-detection threshold; steepness, steepness of the probability curve for delay detection (**p < 0.01; *p < 0.05).

The delay-detection probability curves of the pDCD and TD groups are shown in Figure 3A, while comparative results for Experiment-1 are shown in Figure 3B. Compared to the TD group, the DDT was significantly elevated (*p* < 0.001) and the steepness was significantly flatter (*p* = 0.005) in the pDCD group.

**Figure 4:**
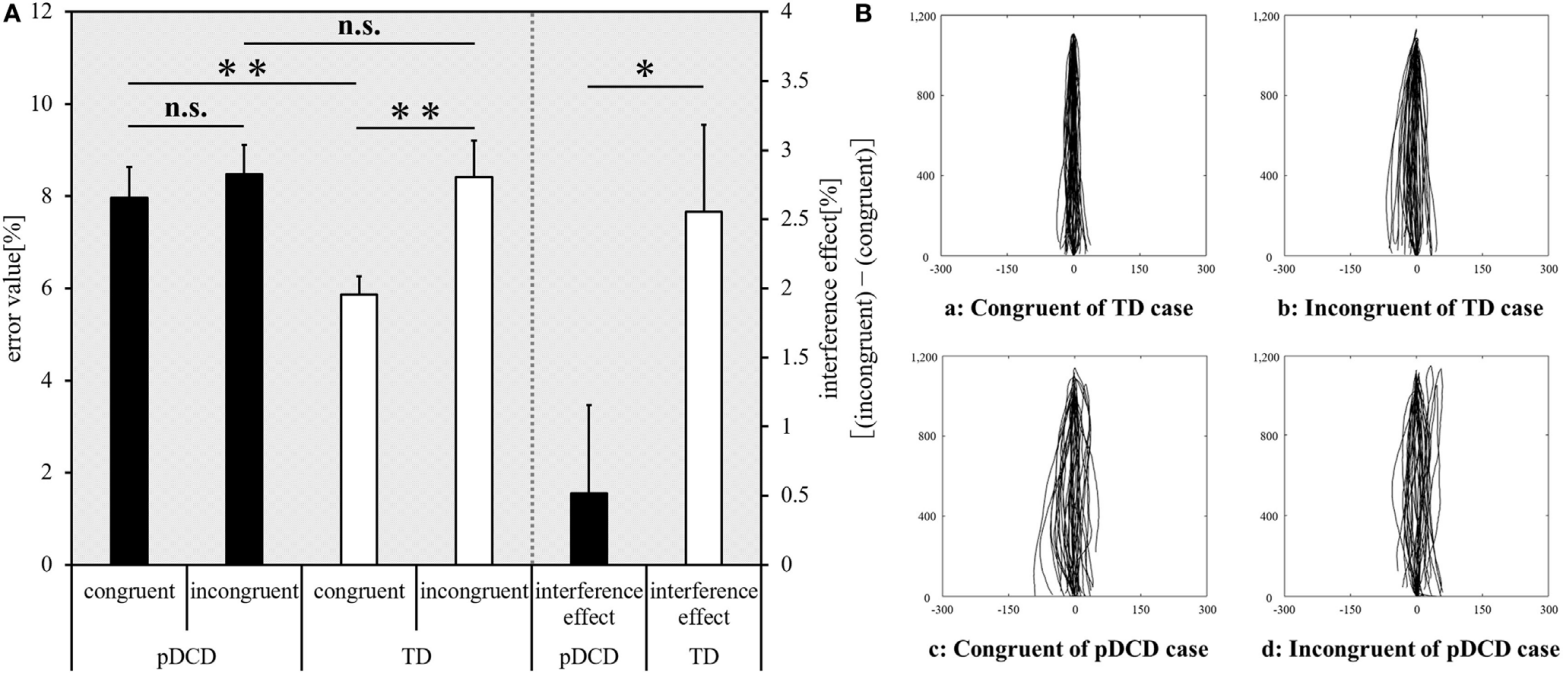
Experiment-2 (Motor interference task) results. **(A)** The mean error value of each condition and interference effect of each group. Horizontal axis shows each condition and interference effect of each group. Black, probable developmental coordination disorder (pDCD) group; white, typical development (TD) group. Error bars represent the SEM. Left, the comparison of the mean error value of each condition in each group; right, the comparison result of the mean interference effect of each group (**p < 0.01; *p < 0.05; n.s. not significant). **(B)** Typical examples of the drawing trajectories of each group. The movement trajectory of a (a) congruent condition and (b) incongruent condition of one example in the TD group (a right-handed 11-year-old boy; manual dexterity = 84th percentile), and a (c) congruent condition and (d) incongruent condition of one example in the pDCD group (a right-handed 11-year-old boy; manual dexterity = 2nd percentile).

The inter-group and intra-group comparison results of Experiment-2 are shown in Figure 4A. Typical examples of the drawing trajectories of each group are shown in Figure 4B. Compared to the TD group, the error value for the congruent condition was significantly increased (*p* = 0.003) and the interference effect was significantly decreased (*p* = 0.044) in the pDCD group. There was no significant difference in the error value during in the incongruent condition between the two groups (*p* = 0.352). Compared to the congruent condition, the error value during the incongruent condition increased in the TD group (*p* < 0.001). In contrast, there was no significant difference in the error value between the congruent and the incongruent conditions in the pDCD group (*p* = 0.094).

**Figure 5:**
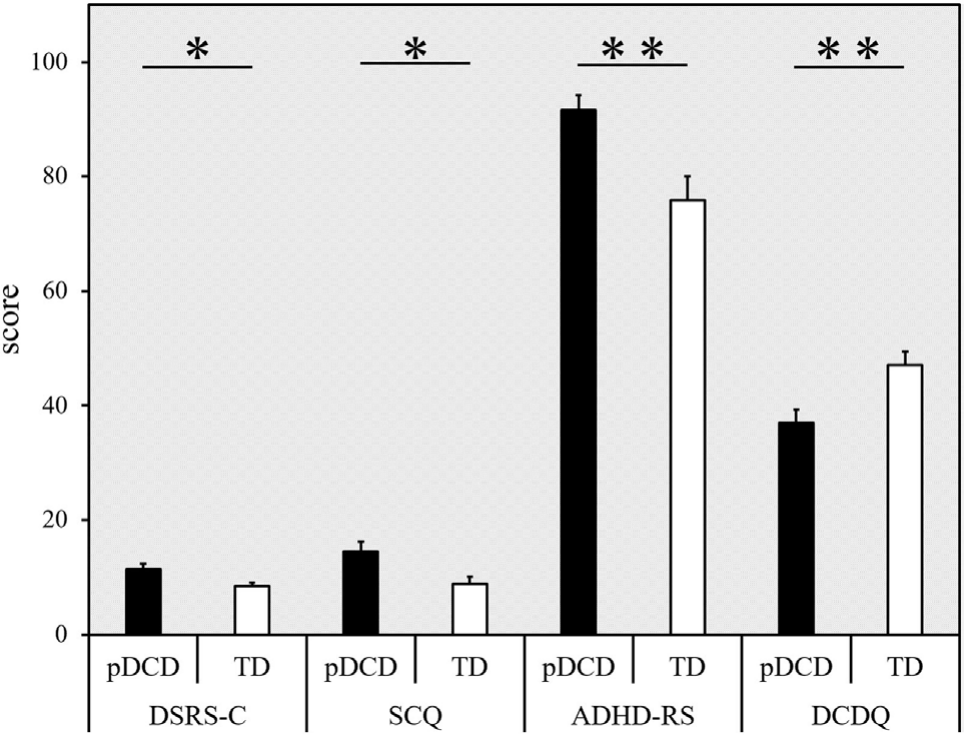
Results from the questionnaires. Score (error bars represent the SEM) for each questionnaire (indicated on the horizontal axis), categorized according to the group. Black, probable developmental coordination disorder (pDCD) group; white, typical developmental (TD) group. DSRS-C, depression self-rating scale for children; SCQ, social communication questionnaire; ADHD-RS, attention-deficit hyperactivity disorder rating scale; DCDQ, developmental coordination disorder questionnaire (**p < 0.01; *p < 0.05).

The inter-group comparison of DSRS-C, SCQ, ADHD-RS, and DCDQ are shown in Figure 5. The DSRS-C score [*t*(69) = 2.551, *p* = 0.013], SCQ score (*p* = 0.012), and ADHD-RS percentile score (*p* = 0.003) were significantly higher in the pDCD group, compared with the TD group. The DCDQ score of the pDCD group was significantly lower than that of the TD group (*p* = 0.009).

### Correlation Analysis Results

The correlation analysis results are shown in Table 1. There was a significant correlation between manual dexterity (percentile), and DDT (*r* = −0.577, *p* < 0.001), steepness (*r* = 0.371, *p* = 0.001) for Experiment-1, error value of the congruent condition for Experiment-2 (*r* = −0.468, *p* < 0.001), SCQ score (*r* = −0.305, *p* = 0.010), ADHD-RS percentile score (*r* = −0.325, *p* = 0.006), and DCDQ score (*r* = 0.356, *p* = 0.002). There was a significant correlation between age, and DDT (*r* = −0.343, *p* = 0.003), steepness (*r* = 0.310, *p* = 0.008), error value of the congruent condition (*r* = −0.451, *p* < 0.001), error value of the incongruent condition (*r* = −0.438, *p* < 0.001), SCQ score (*r* = −0.273, *p* = 0.021), and ADHD-RS percentile score (*r* = −0.240, *p* = 0.044). There was a significant correlation between DDT, and steepness (*r* = −0.435, *p* < 0.001), error value of the congruent condition (*r* = 0.461, *p* < 0.001), error value of the incongruent condition (*r* = 0.238, *p* = 0.046), ADHD-RS percentile score (*r* = 0.329, *p* = 0.005), and DCDQ score (*r* = −0.378, *p* = 0.001). There was a significant correlation between steepness, and error value of the congruent condition (*r* = −0.395, *p* = 0.001), and error value of the incongruent condition (*r* = −0.414, *p* < 0.001). There was a significant correlation between error value of the congruent condition, and error value of the incongruent condition (*r* = 0.706, *p* < 0.001), SCQ score (*r* = 0.349, *p* = 0.003), ADHD-RS percentile score (*r* = 0.281, *p* = 0.018), and DCDQ score (*r* = −0.458, *p* < 0.001). There was a significant correlation between error value of the incongruent condition, and the interference effect (*r* = 0.642, *p* < 0.001), and DCDQ score (*r* = −0.313, *p* = 0.008). There was a significant correlation between SCQ score, and ADHD-RS percentile score (*r* = 0.503, *p* < 0.001), and DCDQ score (*r* = −0.585, *p* < 0.001). There was a significant correlation between ADHD-RS percentile score and DCDQ score (*r* = −0.539, *p* < 0.001).

**Table 1:** Correlation matrix.

	Manual dexterity (percentile)	Age	Experimental task 1		Experimental task 2			DSRS-C	SCQ	ADHD-RS	DCDQ
			DDT	Steepness	Congruent	Incongruent	Interference effect				
Manual dexterity (percentile)	–	0.030	−0.577**	0.371**	−0.468**	−0.219	0.183	−0.196	−0.305**	−0.325**	0.356**
Age		–	−0.343**	0.310**	−0.451**	−0.438**	−0.173	0.032	−0.273*	−0.240*	0.231
**Experimental task 1**											
DDT			–	−0.435**	0.461**	0.238*	−0.150	0.147	0.227	0.329**	−0.378**
Steepness				–	−0.395**	−0.414**	−0.087	−0.057	−0.058	−0.070	0.218
**Experimental task 2**											
Congruent					–	0.706**	0.065	0.073	0.349**	0.281*	−0.458**
Incongruent						–	0.642**	0.016	0.120	0.152	−0.313**
Interference effect							–	−0.083	−0.139	0.014	0.020
DSRS-C								–	0.150	0.137	−0.177
SCQ									–	0.503**	−0.585**
ADHD-RS										–	−0.539**
DCDQ											–

**p < 0.01; *p < 0.05.

*Numbers in the frame show correlation coefficients*.

*ADHD-RS, attention-deficit hyperactivity disorder rating scale; DCDQ, developmental coordination disorder questionnaire; DDT, delay-detection threshold; DSRS-C, depression self-rating scale for children; SCQ, social communication questionnaire; steepness, steepness of the probability curve for delay detection*.

### Multiple Regression Analysis Results

Table 2 shows the results of the multiple regression analysis, where manual dexterity (percentile) was the dependent variable, and DDT/steepness for the Experiment-1, error value of the congruent condition for the Experiment-2, the SCQ score, percentile score of ADHD-RS, and DCDQ score were independent variables. The DDT (β = −0.482, *p* < 0.001) and SCQ score (β = −0.219, *p* = 0.035) were significant independent variables. The following multiple regression equation revealed the relationship between manual dexterity (percentile) and the DDT/SCQ score: manual dexterity (percentile) = 84.747 + (−0.156 × DDT) + (−0.691 × SCQ) (*R* = 0.570, *R*^2^ = 0.325, *p* < 0.001). There was no multicollinearity between the DDT and SCQ scores.

**Table 2:** Multiple regression analysis results.

Dependent variable	Independent variable	Partial regression coefficient [B]	Standardized regression coefficient [β]	*p*-Value	VIF
Manual dexterity [percentile]	(constant)	84.747		<0.001	
	DDT	−0.156	−0.482	<0.001	1.047
	SCQ	−0.691	−0.219	0.035	1.047
	
			*R* = 0.570, *R*^2^ = 0.325, *p* < 0.001		

*DDT, delay-detection threshold; SCQ, social communication questionnaire*.

## Discussion

The participants in the current study were classified as either the pDCD group or TD group, based on if the children had clumsiness of manual dexterity or not, respectively, based on the manual dexterity test of M-ABC2. There were no significant differences in sex, age, and handedness, among the two groups. However, in the pDCD group, the DDT and steepness, which reflected the visuo–motor temporal integration ability, were prolonged and decreased, respectively. In addition, in the pDCD group, the interference effect reflecting the automatic-imitation function was decreased. The correlation analysis showed a significant correlation between manual dexterity (percentile), and DDT/steepness for the delayed visual feedback detection task, error value of the congruent condition for the motor interference task, ASD traits, and ADHD traits. Furthermore, the multiple regression analysis revealed that DDT and ASD traits were significantly predictors for manual dexterity in children.

### Experiment-1: Delayed Visual Feedback Detection Task

The results demonstrated that children with pDCD had a significantly longer DDT and lower steepness of the probability curve for delay detection, compared with children with TD. Thus, children with pDCD had a deficit visuo–motor temporal integration. There was also a significant correlation between manual dexterity (percentile) and DDT/steepness, where a decrease in manual dexterity was associated with a decline in visuo–motor temporal integration ability. Further, DDT was the independent variable that was most predictive of manual dexterity. Although previous studies revealed a deficit in visuo–motor integration in DCD (12), there has been no study that has focused only on the temporal aspect in visuo–motor integration. The delayed visual feedback detection task reflects visuo–motor temporal integration ability, where extended DDT and decline of steepness indicates poor visuo–motor temporal integration. Therefore, the current study supported the IMD hypothesis and provided additional evidence that children with clumsy of manual dexterity have a deficit of visuo–motor temporal integration. DCD is a frequent comorbid disorder of ASD and ADHD. Further, DCD frequently accompanies depressive symptoms. Therefore, we also evaluated ASD traits, ADHD traits, and depression symptoms in the current study. The SCQ is a useful screening test that corroborated well with results from the Autism Diagnostic Interview-Revised and Autism Diagnostic Observation Schedule, which are the gold standards of ASD diagnosis. Further, in children, the ADHD-RS and DSRS-C are useful screening tests for the diagnosis of ADHD and depression, respectively. Thus, it is worth noting that the DDT, which was measured by the delayed visual feedback detection task, was the most significant factor that predicted the severity of clumsiness in children, over these standard evaluation batteries.

An important role of the internal model is to generate error signals between motor predictions and actual sensory feedback, to correct motor commands online. Importantly, error signals can also act as a training signal, refining the accuracy of predictive models. This iterative process is fundamental for motor learning (99). Thus, a major mismatch in motor predictions/actual proprioceptive feedback and actual visual feedback, during the initiation of self-generated movement, can cause unsuccessful movement. In other words, the reduced ability of the visuo–motor temporal integration impedes the generation of error signals, which may cause movement failure. In fact, artificially delaying visual feedback from self-generated hand movement causes a decrease in hand motor performance and hampers adaptive motor learning. Previous studies reported a decrease in motor performance during writing, drawing, star or maze tracing (100), steering (101), manual tracking (102), and pegboard (103), reaching (104), and sequential motor tasks (105), when visual feedback from self-generated movement is substantially delayed. Moreover, studies have demonstrated that a delay in visual feedback slows the rate and extent of prism adaptation (106, 107). Further, another study demonstrated that a delay in visual feedback decreased muscle activity (108). The findings of the current study corroborated with these previous studies, suggesting that a diminished ability to integrate hand movement and visual feedback in a temporal sequence is detrimental to manual dexterity.

Previous studies on adults using the delayed visual feedback detection task, similar to this study, indicated that the parietal cortex and cerebellum were the neural bases for visuo–motor temporal integration (36–39). Further, fMRI studies suggested that the parietal cortex and cerebellar regions may be key structures implementing the comparison between predicted movements and actual sensory feedback that is required for explicit agency judgments (14, 109–111). These previous studies showed that the parietal lobe and cerebellum play a role in the detection of delayed visual feedback from self-generated movement. Therefore, the current results may be interpreted as reflecting parietal and cerebellar dysfunction/developmental failure in children with clumsy manual movements.

### Experiment-2: Motor Interference Task

The error value during the incongruent condition was significantly increased, compared with the congruent condition, in children with TD. However, there were no differences between the congruent condition and the incongruent condition in children with pDCD. Further, in comparison to children with TD, the interference effect was significantly lower in the pDCD group. These results indicated a deficit in automatic imitation in children with clumsy manual dexterity. Previous studies also demonstrated that DCD caused an obstacle to conscious imitation (43–47) due to MNS inactivation (10). The current study corroborated with previous studies and is the first to show a decrease in automatic-imitation function in children with clumsy manual dexterity, compared with children with TD. Automatic imitation is the basic function of the MNS (41, 49–52). The current results strongly suggested that the MNS is dysfunctional in DCD, as the children with pDCD demonstrated deficits in automatic imitation.

### Questionnaires

The DSRS-C score of the pDCD was significantly higher than the TD group, which indicated that children who were clumsy had more depressive symptoms. Poor motor skills are associated with a range of psychosocial issues, including internalizing problems, i.e., anxiety and depression (69). The current study also corroborated with previous studies, which demonstrated that children with DCD have a higher level of depressive symptoms compared with those with TD (68, 71).

The SCQ score of the pDCD group was also significantly higher than the TD group. There was significant correlation between manual dexterity (percentile) and the SCQ score. Furthermore, multiple regression analysis showed that SCQ was a significant independent variable that predicted manual dexterity (percentile). The SCQ is an evaluation battery of social cognitive functions related to ASD. Therefore, the current findings demonstrated that children with pDCD showed high ASD traits, and that increasing ASD traits was associated with worsening manual dexterity. Sumner et al. (78) investigated the extent of overlap of these problems in children aged 7–10 years, who were diagnosed with either ASD or DCD. Compared to the control cohort (children with TD), the motor and social difficulties of children with ASD or DCD showed considerable overlap. Furthermore, they indicated that motor skill predicted social functioning for both ASD and DCD. The present study revealed that social functioning reflected by SCQ predicted motor skill. Other studies also reported that motor function and social cognitive function were correlated in children (112, 113). The current results, in concert with previous studies, demonstrated the important relationship between motor function and social cognitive function in children.

Compared with children with TD, the percentile scores of ADHD-RS was significantly higher in the pDCD group. There was a significant correlation between manual dexterity (percentile) and ADHD-RS score. The connection between ADHD and DCD has been recognized for several decades (74), with an estimated overlap between both disorders of 50% (72, 73, 75). Thus, the current findings showed that children with clumsiness of manual dexterity have high ADHD traits, similar to previous studies.

The DCDQ score of the pDCD group was significantly lower than the TD group. There was a significant correlation between manual dexterity (percentile) and DCDQ score. A previous study showed that the percentile measured by M-ABC2 and the DCDQ score were significantly correlated (96). Since the DCDQ is a subjective assessment of the child’s motor function by the parents, these results reflected the parents’ understanding of their child’s motor ability.

Correlation analysis showed a significant correlation between manual dexterity, and DDT, ASD traits, and ADHD traits. In addition, correlation analysis also showed a significant correlation between DDT and ADHD traits, and between ADHD traits and ASD traits. These results strongly suggested that manual dexterity, time window for visuo–motor integration, ASD traits, and ADHD traits are related to bidirectionality.

### General Discussion

Visuo–motor temporal integration is the ability to integrate self-generated movement and visual information, while automatic imitation also reflects the integrated function of self and other movements. Thus, both are a reflection of visuo–motor integration. Thus, the present study revealed that children with clumsy manual dexterity, i.e., pDCD, had deficits in visuo–motor integration. The parietal lobes and cerebellum are the neural bases of visuo–motor temporal integration and automatic imitation. Thus, dysfunction of these brain regions may lead to difficulties in visuo–motor integration, which in turn could lead to the development of clumsiness in children.

However, multiple regression analysis highlighted visuo–motor temporal integration (DDT), but not automatic imitation, as a significant predictor of manual dexterity in children. This may be due to the influence of the characteristics of the two experimental tasks, where the delayed visual feedback detection task was a cognitive task that required judgment. The motor interference task, however, reflected automatic imitation, which does not require cognitive function. Many previous studies have reported that the child’s manual dexterity ability is related to cognitive functions, such as math, reading, and science (114–116). The correlation between manual dexterity and cognitive processing is observed both in children with DCD and in children without DCD (117). Therefore, although this explanation is speculative, DDT obtained by the delayed visual feedback detection task, which required cognitive function, may more accurately predict manual dexterity.

In conclusion, compared with the TD group, the pDCD group showed significant deficits in visuo–motor temporal integration and automatic imitation. Thus, the results of this study concurrently supported both the IMD hypothesis and MNS deficits hypothesis. However, given that multiple regression analysis revealed that the index of visuo–motor temporal integration ability was the most important predictor for the clumsiness of manual dexterity, we strongly support the IMD hypothesis.

### Limitations of the Current Study

The current study had several limitations, which must be noted. The pDCD group in the current study did not completely satisfy the DCD diagnostic criterion A in DSM-5. The DCD diagnostic criteria A in DSM-5 requires a M-ABC2 total test score that is in or below the 15th percentile. However, the criteria in the current study required an M-ABC2 manual dexterity test component score in or below the 15th percentile because this study specifically focused on manual dexterity. Therefore, the current results are limited to the results of children with difficulty of manual dexterity. Further research by participants who fully satisfy the DSM-5 DCD diagnostic criteria A through D is necessary.

The study focused on the children’s manual dexterity, classified the children with pDCD based on manual dexterity, and revealed their deficits in visuo–motor temporal integration ability and automatic-imitation function. The relationship between other movement dysfunctions that are observed with DCD, i.e., gross motor skills and balance disorders, and visuo–motor temporal integration and automatic imitation, however, are unknown.

In the current study, two experimental tasks showed that children with pDCD have deficits in visuo–motor temporal integration and automatic imitation. However, the findings of both tasks of delayed visual feedback detection task and motor interference task may be explained by the automatization deficit, attention deficit, and timing deficit hypotheses. In automatization deficit hypothesis, a dual task of motor and cognitive function is generally used, which in the past has been verified as the etiology of developmental dyslexia (118), and recently was suggested as a pathogenesis of DCD (119). The attention-deficit hypothesis has been discussed in the context of ADHD (120). The timing deficit hypothesis, which has been mainly verified as the etiology of ADHD (121), has also been suggested to underlie DCD (122). However, several studies investigating the automatization deficit of DCD also reported negative results (16, 123–127). We also evaluated ADHD traits (using ADHD-RS), which were previously shown to be strongly related to the attention-deficit and timing deficit hypotheses, but were not a significant predictor of manual dexterity in the current study. Therefore, we believe that the current results cannot really be attributed to either of these hypotheses. However, further research is required to verify these findings, using a new experiment method that controls for dual task conditions, attention, and timing.

This study also did not measure the intelligence quotient (IQ) of children. Thus, there is a possibility that IQ may have affected the current results. However, the participants in this study attended regular classes at public preschools, public primary schools, or public secondary schools, and they had not been diagnosed with an intellectual disability. Thus, IQ was assumed to have no effect on the current results. Nonetheless, it remains to be measured in a future study to yield more definitive conclusions.

Self-generated movement has two components, motor predictions and proprioceptive feedback. Thus, whether visuo–motor temporal integration as was measured in this study indicates the ability to integrate motor predictions and visual feedback or the ability to integrate proprioceptive feedback and visual feedback is not clear. A future study needs to clearly contrast passive movement (a task to detect delayed visual feedback from proprioception) and active movement (a task to detect delayed visual feedback from active movement, which was used in this study).

### Future Directions

Although deficits in the cerebellum (28, 29, 33, 34) and parietal lobe (29–34) are thought to underlie DCD, this has not yet been confirmed (128). Therefore, visuo–motor temporal integration may serve as psychophysical marker of DCD in future neuroimaging studies that evaluate the neural signature of DCD.

The current study suggested that improving visuo–motor temporal integration may be effective as rehabilitation for DCD. Therefore, it is necessary to develop new neurorehabilitation techniques that will promote visuo–motor temporal integration. We specifically focused on rehabilitation using stochastic resonance. Stochastic resonance is a phenomenon in which random frequency noise, which is less than or equal to the sensory threshold, is added to the body, the rhythm activity in the peripheral or central nervous system is superimposed on noise and the sensitivity of the somatosensory sense increases. Therefore, it might be hypothesized that stochastic resonance may improve the visuo–motor temporal integration function and motor function of children with DCD. This hypothesis needs to be investigated by a randomized controlled trial of children with DCD.

## Ethics Statement

The experimental procedures were approved by the local ethics committee of the Graduate School and Faculty of Health Sciences at Kio University (approval number: H27-33). There were no foreseeable risks to the participants; no personally identifying information was collected. Participants (i.e., each child’s parents) provided background information and gave written informed consent. The procedures complied with the ethical standards of the 1964 Declaration of Helsinki regarding the treatment of human participants in research.

## Author Contributions

SN collected and analyzed the data, and wrote the manuscript. AS, TT, TS, and EF assisted in collecting data. SN designed the study. YN, DA, EF, MO, SS, SM, and AN designed and supervised the study. All authors read and approved the manuscript.

## Conflict of Interest Statement

The authors declare that the research was conducted in the absence of any commercial or financial relationships that could be construed as a potential conflict of interest.
